# Liver Disease-Related Sarcopenia: A Predictor of Poor Prognosis by Accelerating Hepatic Decompensation in Advanced Chronic Liver Disease

**DOI:** 10.7759/cureus.49078

**Published:** 2023-11-19

**Authors:** Atsushi Nakamura, Tsubasa Yoshimura, Takeshi Ichikawa

**Affiliations:** 1 Hepatology, Nippon Koukan Hospital, Kawasaki, JPN; 2 Gastroenterology, Nippon Koukan Hospital, Kawasaki, JPN

**Keywords:** hyponatremia, ascites, portal hypertension, sarcopenia, magnetic resonance elastography

## Abstract

Background

Sarcopenia is considered a prognostic factor for advanced chronic liver disease (ACLD) independent of liver function, but the underlying mechanisms are unknown. Here, we investigated whether sarcopenia contributed to hepatic decompensation and worsened prognosis.

Methods

This was a single-center retrospective study of 708 patients with chronic liver disease who underwent magnetic resonance elastography (MRE). Magnetic resonance imaging (MRI) was used to diagnose sarcopenia and hepatic decompensation (presence of ascites).

Results

The incidence of sarcopenia (29% overall) and age were significantly correlated to increased liver stiffness (LS) (p < 0.01 each), but age did not differ for LS ≥ 4 kPa. Rates of thrombocytopenia and varices increased at ≥4 kPa, and ascites (n = 52) accounted for 81% of patients with ≥6 kPa LS. Age, alcoholic liver disease, C-reactive protein, sodium level, and controlling nutritional status score were extracted as factors contributing to sarcopenia (all p < 0.05). In ACLD, sarcopenia was an independent predictor of ascites (p < 0.01), and in a follow-up analysis of 163 patients without ascites at baseline, the incidence of ascites in patients with sarcopenia was significantly higher, even after adjusting for LS and liver severity (p < 0.01). The Cox proportional hazards model indicated albumin-bilirubin score and sarcopenia as independent prognostic factors (p < 0.01 each).

Conclusions

In ACLD, both portal hypertension and liver disease-related sarcopenia were found to occur at ≥4 kPa. Sarcopenia was accompanied by mildly decreased sodium levels and contributed to the early development of ascites and poor prognosis, independent of liver function.

## Introduction

Liver fibrosis, the most critical prognostic factor in chronic liver disease (CLD), has been shown to significantly impact patient outcomes [1,2]. Magnetic resonance elastography (MRE) is currently considered the premier noninvasive test (NIT) for the diagnosis and staging of liver fibrosis, supported by numerous studies in various etiologies [3,4]. On the other hand, there is a significant a disparity in survival rates between the compensated and decompensated phases of cirrhosis, with liver stiffness (LS) on MRE demonstrating superior ability to assess the risk of hepatic decompensation and mortality in cirrhosis [5,6].

Sarcopenia (i.e., loss of muscle mass), a phenotype of malnutrition, has been reported to be associated with poor prognosis in cirrhosis, independent of hepatic reserve function score [7,8]. However, the underlying mechanisms are complex and not yet fully understood. One of our clinical questions about sarcopenia in cirrhosis is why the prognostic impact of sarcopenia on patients with cirrhosis has only been shown in Child-Pugh class A or B [9]. Therefore, we sought to investigate the relationship between sarcopenia and hepatic decompensation. We believe that one of the reasons for the difficulty in elucidating the mechanisms of sarcopenia in CLD is that previous reports have analyzed the factors contributing to sarcopenia without distinguishing between aging and diseases.

Insulin-like growth factor-1 (IGF-1) is a hormone synthesized mainly in the liver (80%) under the control of growth hormone (GH) [10]. IGF-1 then stimulates mammalian target of rapamycin (mTOR) to promote muscle protein synthesis and simultaneously inhibits myostatin, a negative regulator of satellite cells, and these two actions promote muscle growth [11]. Therefore, analysis of the relationship between IGF-1 levels and LS may help understand the pathogenesis of sarcopenia associated with liver disease. In addition, magnetic resonance imaging (MRI) has recently been successfully used to diagnose sarcopenia [12,13].

The aim of this study was to investigate our hypothesis that liver disease-related sarcopenia promotes hepatic decompensation and worsens prognosis in advanced chronic liver disease (ACLD) by analyzing MRE imaging data.

## Materials and methods

Study population

In this study, 708 of 714 patients who underwent MRE at Nippon Koukan Hospital in Japan from July 1, 2017, to July 31, 2022, were included in the analysis. MRE was conducted to diagnose liver fibrosis and hepatic steatosis, as well as the presence of liver tumors on non-contrast MRI. The six patients excluded from the analysis included two who had undergone splenectomy and four who had difficulty with elastography due to massive ascites or excessive iron deposits. The CLD etiologies were hepatitis B, hepatitis C, non-alcoholic fatty liver disease (NAFLD), alcoholic liver disease (ALD), autoimmune liver disease, and unknown cause in 143, 165, 219, 93, 47, and 41 patients, respectively. All hepatitis C patients who achieved sustained viral response (SVR) with treatment were included.

Our study was approved by the Ethical Review Board of Nippon Koukan Hospital (Approval No. 202014). The study was conducted in accordance with the "Ethical Principles for Medical Research Involving Human Subjects" described in the 1975 Declaration of Helsinki (revised in 2000). Informed consent of participants was obtained in an opt-out manner.

Diagnostic criteria for the etiology of chronic liver disease, cirrhosis, and hepatocellular carcinoma

ALD was diagnosed according to the diagnostic criterion of the Japanese Society for Biomedical Research on Alcohol [14], and NAFLD was diagnosed according to imaging findings (hepatic and renal contrast on abdominal ultrasonography; liver/spleen ratio < 0.9 on abdominal computed tomography (CT); MRI-proton density fat fraction [PDFF] > 5.2% [15]) and lack of alcohol overuse (pure ethanol equivalent of <30 and <20 g/day for males and females, respectively). Cirrhosis was diagnosed based on histological or clinical findings. Clinical findings include typical ultrasound findings, low platelet counts (<100,000/μL), and complications such as varices. Hepatocellular carcinoma (HCC) was diagnosed according to the guidelines [16,17], as tumors marked in the arterial phase on contrast-enhanced CT and showing washout in the portal or delayed phase. In the case of gadoxetate disodium contrast-enhanced MRI, tumors were also defined as those that stained in the early phase and showed washout in the portal venous phase.

Detailed data collection instructions

Parameters of age, gender, height, weight, body mass index (BMI), and baseline laboratory data were collected for each patient. The mean and median intervals between clinical data and MRE dates used in the analysis were 9 ± 10 days and 7 (0-16) days, respectively. ACLD in this study was defined as F3 and F4 patients diagnosed by MRE, and the severity of liver dysfunction was assessed by albumin-bilirubin (ALBI) score [18] and model for end-stage liver disease sodium (MELD-Na) score [19]. In addition, F4 patients were stratified by modified albumin-bilirubin (mALBI) grade [20], and ACLD was divided into five stages (F3 to mALBI grade G3).

MRE protocol

In our institution, MRE was performed as an add-on to a conventional non-contrast MRI examination. All patients fasted overnight (>12 hours) before being evaluated using a 1.5-T whole-body MRI system (SIGNA Voyager XT 1.5T; GE Medical Systems, Milwaukee, WI, USA). LS was measured via elastography to determine hepatic fibrosis progression [21], whereas intrahepatic fat content was measured using IDEAL IQ as PDFF [22]. The respective measurements of LS (kPa) and PDFF (%) were analyzed by a radiologist skilled in liver imaging. The diagnosis of F stage was made according to two criteria for each etiology, considering that liver stiffness measurement (LSM) on MRE differed according to etiology. NAFLD was diagnosed using the criteria of Hsu et al. (fibrosis stages 0 through 4, with thresholds of 2.61 kPa, 2.97 kPa, 3.62 kPa, and 4.69 kPa, respectively) [23]. The other causes were diagnosed using Morisaka et al.’s criteria (threshold values of 2.32 kPa, 2.61 kPa, 3.02 kPa, and 4.23 kPa, respectively) [24].

Nutritional assessment

In this study, malnutrition was assessed using two methods. First is the CONUT (controlling nutritional status) score [25] calculated from three blood tests, and the second is sarcopenia diagnosed by MRI imaging. Skeletal muscle mass was measured as previously reported [12,13]. In practice, paraspinal muscle area (PSMA) was measured at the level of the origin of the superior mesenteric artery using MRI images and the PSM index (PSMI) corrected by the square of height. In practice, PSMA was measured at the level of the origin of the superior mesenteric artery using MRI images and PSMI corrected by the square of height. In addition, serum IGF-1 levels (ng/mL) in CLD were also measured in 242 patients without diabetes medication or insulin therapy. Because serum IGF-1 levels are sex-dependent and impacted by age, measured serum IGF-1 levels were calculated as %IGF-1 from the ratio of median serum IGF-1 levels (for each sex) for each age group (0-77 years) in a healthy Japanese population [26]. In patients aged 78 years or older, IGF-1 levels were corrected for the median serum IGF-1 level at the age of 77 years.

Assessment of portal hypertension

Thrombocytopenia (platelet count <15x104/mm3) [27,28], and gastric or esophageal varices were used to evaluate portal hypertension in this study. Varices were diagnosed by esophagogastroduodenoscopy (EGD) performed within one year after MRE (mean interval: 70±79 days, median: 53 days). Hepatic decompensation was diagnosed by ascites (≥grade 1) on MRI imaging [7]. ACLD was defined as hepatic fibrosis stage 3 or 4 by MRE. The cumulative incidence of ascites was then analyzed in 162 patients with ACLD without ascites at baseline who could be followed up with contrast CT or MRI imaging (mean observation period: 24.7 ± 12.7 months, median: 26.9 months).

Statistical analysis

JMP statistical software (version 12.2, SAS Institute Japan, Tokyo, Japan) was used for all statistical analyses. Chi-square test, Wilcoxon-Mann-Whitney test, and Spearman’s rank correlation coefficient were used for inter-group analyses, and multiple comparisons of variables among multiple groups were performed using the Steel-Dwass post-hoc or Steel tests after confirming significant differences by the Kruskal-Wallis test. Logistic regression analysis was used to examine factors associated with ascites in ACLD. A stepwise increase/decrease method was used to select variables. Patient prognosis was analyzed using Kaplan-Meier and Cox proportional hazards methods, and the stepwise decrease method was used for variable selection. Statistical significance was set at a p-value of <0.05.

## Results

Baseline characteristics

The characteristics of all patients are shown in Table 1. The median age of all patients was 62 years (range: 15 to 94), and sarcopenia was present in 203 (29%) patients. Patients with sarcopenia were older and had a lower body mass index (BMI) than those without sarcopenia. Hepatitis C and ALD were the most common causes. In addition, patients with sarcopenia were more likely to have advanced stages of liver fibrosis (F3/F4: 59% vs. 30%, p < 0.05) and had significantly higher rates of C-reactive protein (CRP) (≥0.5 mg/dL) positivity, portal hypertension, and HCC. 

**Table 1:** Clinical characteristics at baseline Statistics are shown as mean ± SD (standard deviation) or n (%). ALBI, albumin–bilirubin grade; ACLD, advanced chronic liver disease; ALD, alcoholic liver disease; ALP, alkaline phosphatase; ALT, alanine aminotransferase; AST, aspartate aminotransferase; BMI, body mass index; BUN, blood urea nitrogen; CONUT, controlling nutritional status; CRP, C-reactive protein; γ-GTP, γ-glutamyl transpeptidase; HCC, hepatocellular carcinoma; IGF-1, insulin-like growth factor-1; LS, liver stiffness; MELD-Na, model for end-stage liver disease sodium; MRE, magnetic resonance elastography; NAFLD, non-alcoholic fatty liver disease; NLR, neutrophil-to-lymphocyte ratio; PT, prothrombin activity; TC, total cholesterol

	All cases (708)	Non-sarcopenia (505)	Sarcopenia (203)	p-value
Age (years)	62 ± 15	60 ± 14	69 ± 13	<0.001
Sex (M/F)	421/287	289/216	132/71	0.724
BMI (kg/m^2^)	24.5 ± 4.3	25.3 ± 3.9	22.6 ± 3.9	<0.001
Etiology of liver disease				
NAFLD	219 (31)	192 (38)	27 (13)	
Hepatitis B	143 (20)	107 (21)	36 (18)	
Hepatitis C	165 (23)	108 (21)	57 (28)	
ALD	93 (13)	39 (8)	54 (27)	
Others	88 (13)	59 (12)	29 (14)	
Laboratory data				
Leukocytes (/mm^3^)	5782 ± 1716	5928 ± 1559	5242 ± 2014	<0.001
Hemoglobin (g/dL)	13.8 ± 1.9	14.2 ± 1.8	12.9 ± 2.0	<0.001
Platelets (×10^4^/mm^3^)	20.1 ± 6.7	21.2 ± 6.2	17.4 ± 7.1	<0.001
Total bilirubin (mg/dL)	1.0 ± 0.9	0.9 ± 0.7	1.3 ± 1.3	<0.001
AST (IU/L)	41 ± 84	45 ± 111	43 ± 57	0.165
ALT (IU/L)	48 ± 148	54 ± 168	33 ± 54	<0.001
ALP (IU/L)	284 ± 181	259 ± 129	342± 261	<0.001
γ-GTP (IU/L)	95 ± 177	86 ± 150	117 ± 229	0.762
Albumin (g/dL)	4.1 ± 0.5	4.2 ± 0.4	3.8 ± 0.7	<0.001
TC (mg/dL)	193 ± 42	199 ± 36	176 ± 49	<0.001
Triglyceride (mg/dL)	148 ± 116	156 ± 123	123 ± 95	<0.001
BUN (mg/dL)	16 ± 8	15 ± 6	18 ± 12	<0.01
Creatinine (mg/dL)	0.8 ± 0.7	0.8 ± 0.2	1.0 ± 0.8	0.187
Sodium (mEq/L)	140 ± 3	141 ± 2	139 ± 4	<0.001
PT (%)	97 ± 17	99 ± 17	91 ± 18	<0.001
Ammonia (μg/dL)	37 ± 29	33 ± 24	43 ± 38	0.022
CRP (≥0.5 mg/dL)	11 %	6 %	26 %	0.002
%IGF-1	72 ± 32	76 ± 32	63 ± 31	0.007
NLR	1.94 ± 1.08	1.78 ± 0.82	2.35 ± 1.51	<0.001
LS (kPa)	3.52 ± 2.13	3.17 ± 1.76	4.42 ± 2.66	0.005
Fibrosis classification by MRE (F0-2/3-4)	440/268	356/149	84/119	0.002
HCC	60 (8)	27 (5)	33 (16)	<0.001
Varices	76/424 (18)	29/286 (10)	47/138 (34)	<0.001
Ascites	52 (7)	10 (2)	42 (21)	<0.001
Diuretics	35 (5)	4 (1)	31 (15)	<0.001
CONUT score	1.3 ± 2.1	0.8 ± 1.3	2.7 ± 2.8	<0.001
ALBI score (ACLD)	–2.24 ± 0.68	–2.20 ± 0.46	–2.41 ± 0.72	<0.001
MELD-Na score (ACLD)	9 ± 6	9 ± 6	8 ± 5	0.081

LS and sarcopenia in CLD

**Figure 1:**
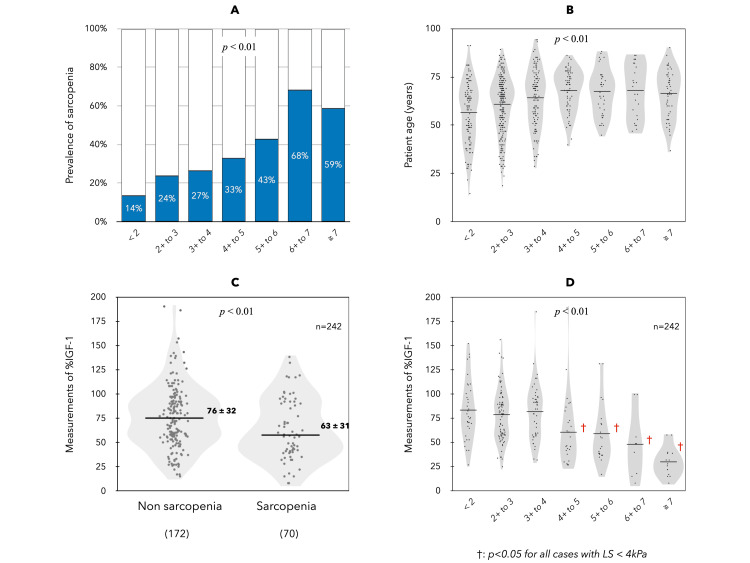
Sarcopenia, age, and %IGF-1 in CLD compared in seven groups based on LS (<2, 2+ to 3, 3+ to 4, 4+ to 5, 5+ to 6, 6+ to 7, ≥7 kPa) The mean LS measured by MRE in CLD was 3.49 ± 2.13 kPa, and patients were classified into seven groups (<2, 2+ to 3, 3+ to 4, 4+ to 5, 5+ to 6, 6+ to 7, and ≥7 kPa) based on LS. The number of patients in each group was 118, 274, 128, 70, 42, 25, and 51, respectively. CLD, chronic liver disease; IGF-1, insulin-like growth factor-1; LS, liver stiffness

The mean LS measured by MRE in CLD was 3.49 ± 2.13 kPa, and patients were classified into seven groups (< 2, 2+ to 3, 3+ to 4, 4+ to 5, 5+ to 6, 6+ to 7, and ≥7 kPa) based on LS. The number of patients in each group was 118, 274, 128, 70, 42, 25, and 51, respectively. In CLD, sarcopenia (29% overall) was significantly associated with higher LS (Figure 1A). The mean age of each group was positively correlated with increasing LS, but the age difference disappeared at LS ≥ 4 kPa (Figure 1B). In 242 CLD cases, %IGF-1 was analyzed; patients with sarcopenia had significantly lower %IGF-1 levels than those without sarcopenia (Figure 1C). The %IGF-1 levels of the seven groups classified by LS showed no change when LS was <4 kPa and significantly decreased when LS was ≥4 kPa (Figure 1D).

Prevalence of liver-related events associated with elevated LS in CLD

**Figure 2:**
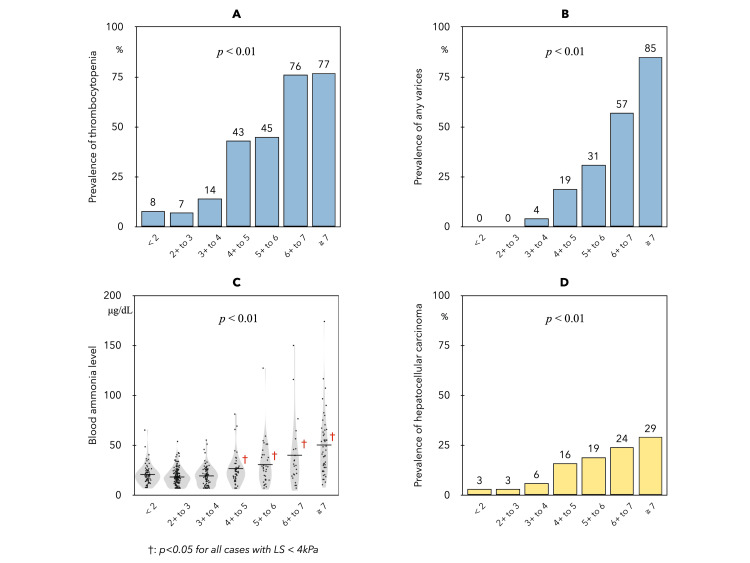
Prevalence of portal hypertension in each of the seven groups based on LS. Thrombocytopenia (A) was significantly increased in patients with LS ≥ 4 kPa (< 4 kPa vs. 4+ to 5 kPa, 9% vs. 43%), and the prevalence of varices (B) was also increased in patients with LS ≥ 4 kPa (1% vs. 19%) (p<0.01 for each).  Serum ammonia levels (C) in the seven LS groups were 30 ± 15, 27 ± 13, 28 ± 15, 39 ± 26, 45 ± 33, 59 ± 52, and 75 ± 46, respectively. LS, liver stiffness

The prevalence of thrombocytopenia (Figure 2A) and any varices (Figure 2B) both showed an increase in patients with LS ≥4 kPa. In addition, serum ammonia levels (Figure 2C) in the seven LS groups were also significantly increased in patients with LS ≥4 kPa. The HCC complication rate (Figure 2D) showed a significant modest linear correlation with increased LS.

Multivariate analysis of factors associated with sarcopenia in CLD

Factors that were significant in the univariate analysis were used in the multivariate analysis. The analysis was performed on nine factors selected by stepwise logistic regression (Table 2). The results showed that age, BMI, ALD, CRP, total cholesterol, and serum sodium level were independent factors associated with sarcopenia in patients with CLD.

**Table 2:** Risk factors associated with sarcopenia in CLD. ALT, alanine aminotransferase; BMI, body mass index; BUN, blood urea nitrogen; CLD, chronic liver disease; CRP, C-reactive protein; NLR, neutrophil-to-lymphocyte ratio;  TC, total cholesterol

	Univariate analysis		Multivariate analysis	
	Odds ratio (95% CI)	p-Value	Odds ratio (95% CI)	p-Value
Age (years)	1.05 (1.04–1.07)	<0.001	1.05 (1.03–1.07)	<0.001
BMI (kg/m^2^)	0.82 (0.79–0.87)	<0.001	0.81 (0.75–0.88)	<0.001
Etiology (alcohol)	4.33 (2.77–6.84)	<0.001	2.79 (1.45–5.35)	0.002
ALT (IU/L)	0.99 (0.98–0.99)	0.002	0.99 (0.98–0.99)	0.006
TC (mg/dL)	0.99 (0.98–0.99)	<0.001	0.99 (0.98–0.99)	0.036
Sodium (mEq/L)	0.83 (0.77–0.89)	<0.001	0.88 (0.79–0.97)	0.007
CRP (mg/dL)	3.62 (2.23–6.32)	<0.001	2.49 (1.16–15.93)	0.016
NLR	1.61 (1.37–1.92)	<0.001	0.98 (0.78–1.23)	0.864
Diuretics	22.57 (8.78–76.70)	<0.001	2.34 (0.66–9.94)	0.196

As an additional analysis (Table 3), univariate analysis of factors associated with %IGF-1 levels was performed separately for patients with LS ≥ 4kPa and LS < 4kPa. The results showed that albumin, total cholesterol, total bilirubin, sodium, prothrombin time, ammonia, CRP, and hepatic reserve function scores were each significantly associated with %IGF-1 in patients with LS≥4kPa (p<0.05 for each).

**Table 3:** Correlation between %IGF-1 values and liver function data in CLD (n=242) ALBI, albumin-bilirubin; ALP, alkaline phosphatase; ALT, alanine aminotransferase; AST, aspartate aminotransferase; BUN, blood urea nitrogen; CLD, chronic liver disease; CRP, C-reactive protein; CONUT, controlling nutritional status; CRP, C-reactive protein; γ-GTP, γ-glutamyl transpeptidase; IGF-1, insulin-like growth factor-1; LS, liver stiffness; TG, triglycerides

	LS < 4 kPa (N=168)		LS ≥ 4 kPa (N=74)	
	r	p-Value	r	p-Value
Age (years)	-0.013	0.870	0.041	0.725
Total bilirubin (mg/dL)	0.014	0.859	-0.456	<0.001
AST (IU/L)	-0.084	0.637	0.045	0.637
ALT (IU/L)	0.071	0.361	0.186	0.103
ALP (IU/L)	0.108	0.279	-0.349	0.007
γ-GTP (IU/L)	0.060	0.448	0.020	0.878
Alb (g/dL)	0.016	0.836	0.566	<0.001
Total cholesterol (mg/dL)	0.213	0.008	0.376	<0.001
TG (mg/dL)	0.055	0.485	0.275	0.019
BUN (mg/dL)	0.045	0.688	-0.010	0.938
Creatinine (mg/dL)	-0.040	0.610	-0.102	0.384
Leucocytes (/mm^3^)	0.028	0.725	0.024	0.592
Hemoglobin (g/dL)	0.032	0.679	0.119	0.314
Platelets (/mm^3^)	0.174	0.024	0.208	0.073
Prothrombin activity (%)	0.071	0.381	0.491	<0.001
Ammonia (μg/dl)	-0.215	0.012	-0.571	<0.001
Sodium (mEq/L)	0.052	0.505	0.337	<0.001
CRP (mg/dL)	0.017	0.847	-0.423	<0.001
LS (kPa)	-0.076	0.329	-0.399	<0.001
ALBI score	-0.007	0.923	-0.594	<0.001
CONUT score	-0.111	0.152	-0.506	<0.001

Analysis of factors associated with ascites complications in ACLD (n=268)

**Figure 3:**
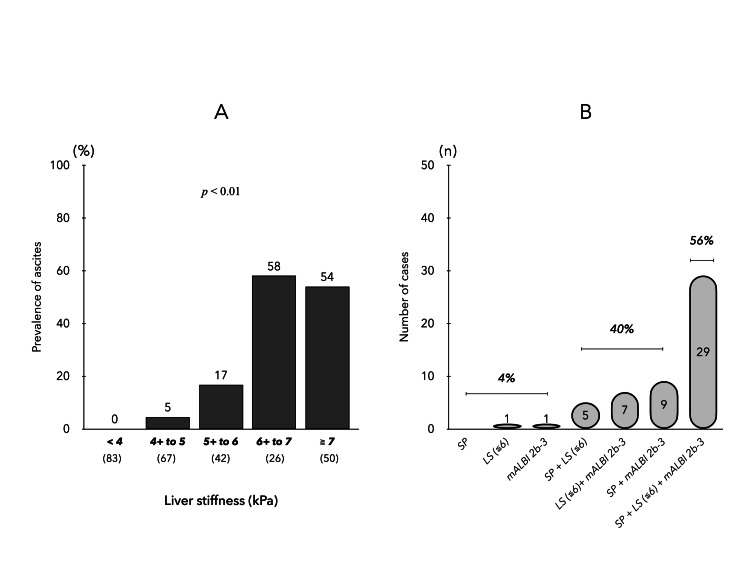
Complications of ascites in patients with ACLD (A) Ascites complication rate in five groups of ACLD patients (based on LS values). (B) Overlapping prevalence of three factors: sarcopenia, LS (≥ 6 kPa), and mALBI grade (2b-3) in patients with ascites. ACLD, advanced chronic liver disease; LS, liver stiffness; mALBI, modified albumin-bilirubin

In ACLD, there were 52 cases of ascites complications. Comparison of ascites complication rates in the five groups based on LS values showed that LS ≥ 6 kPa resulted in increased ascites (Figure 3A). Univariate analysis of factors associated with ascites was performed for age, gender, BMI, etiology, blood parameters, LS, neutrophil-to-lymphocyte ratio, model for end-stage liver disease-sodium (MELD-Na) score, ALBI score, presence of varices, sarcopenia, and HCC. In a multivariate analysis with six significant factors selected by the stepwise method (Table 4), LS ≥ 6 kPa, ALBI score, and sarcopenia were independent associated factors for ascites in ACLD. A study of these three factors in patients with complicated ascites showed a prevalence of LS ≥ 6 kPa in 81%, mALBI grade 2b/3 in 89%, and sarcopenia in 83%, with 96% of patients with complicated ascites having two or more factors (Figure 3B).

**Table 4:** Risk factors associated with ascites in ACLD ACLD, advanced chronic liver disease; ALBI, albumin–bilirubin; LS, liver stiffness

	Univariate analysis		Multivariate analysis	
	Odds ratio (95% CI)	p-Value	Odds ratio (95% CI)	p-Value
Triglyceride (mg/dL)	0.98 (0.97–0.99)	<0.001	0.99 (0.97–1.00)	0.133
Ammonia (μg/dL)	1.03 (1.02–1.04)	<0.001	1.01 (0.99–1.04)	0.192
LS (≥6 kPa)	21.32 (10.13–48.80)	<0.001	6.61 (1.34–44.07)	0.020
Varices (any)	22.04 (9.24–61.70)	<0.001	3.89 (0.80–21.27)	0.122
Sarcopenia	13.24 6.33–61.70)	<0.001	8.11 (1.74–51.92)	0.007
ALBI score	27.26 (12.24–71.28)	<0.001	7.96 (2.02–49.34)	0.002

Prospective study of new occurrence of ascites in patients with ACLD

**Figure 4:**
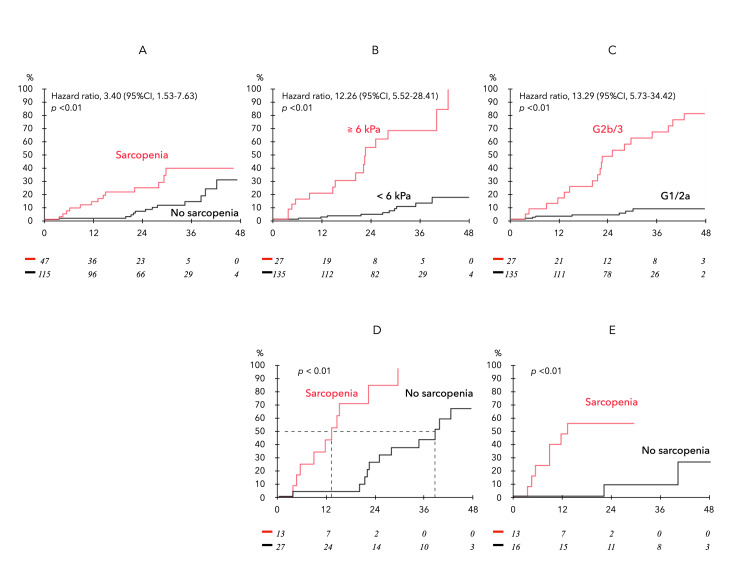
Analysis of the cumulative incidence of ascites in 162 subjects without ascites at baseline. New ascites onset occurred in 27 patients during the follow-up period. (A) Comparison by the presence/absence of sarcopenia. (B) Comparison by LSM ≥ 6.0 kPa and < 6.0 kPa. (C) Comparison by mALBI grade 2b/3 and 1/2a. (D) Comparison by the presence/absence of sarcopenia in patients with LS ≥ 6.0 kPa or mALBI grade 2b/3. The median event-free period was 14 and 38 months, respectively. (E) The incidence of new ascites in HCC patients (n=29). HCC, hepatocellular carcinoma; LSM, liver stiffness measurement; mALBI, modfied albumin–bilirubin

During the follow-up of 162 patients without ascites at baseline, 27 patients developed new ascites (observation period 24.7 ± 12.7 months). Analysis of the cumulative incidence of ascites by sarcopenia (Figure 4A), LS≥ 6kPa (Figure 4B), and mALBI grade 2b-3 (Figure 4C) showed a significant difference in the incidence of ascites with and without each factor by Cox proportional hazards model. Furthermore, Figure 4D shows the incidence of new ascites in patients with LS ≥ 6.0 kPa or mALBI grade 2b-3 (n=40) with and without sarcopenia, with the sarcopenia group showing an earlier occurrence of ascites (median event-free period with and without sarcopenia of 14 and 38 months, respectively). A similar trend was observed in HCC patients (n=29) (Figure 4E).

Impact of sarcopenia on prognosis in ACLD

**Figure 5:**
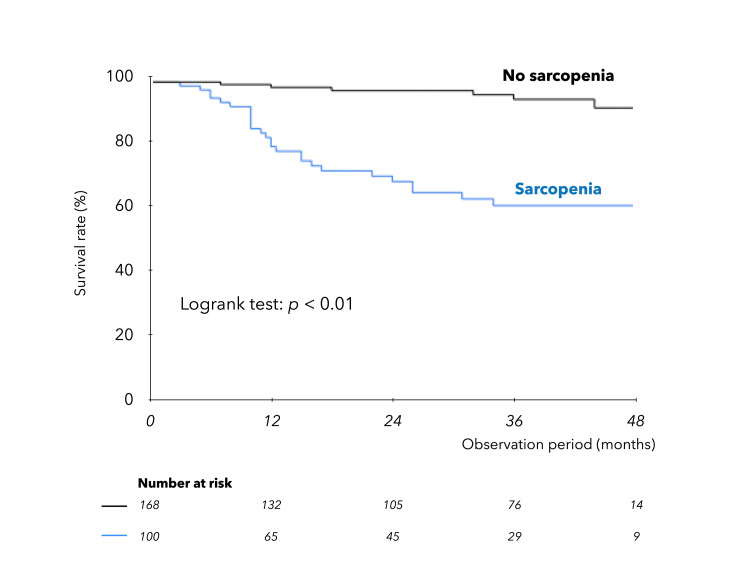
Kaplan-Meier survival curves in ACLD. Comparison of survival of patients with and without sarcopenia complications. ACLD, advanced chronic liver disease

During a mean interval of 27.1 ± 15.8 months (median: 29 months), 38 liver-related deaths occurred, including two related to liver transplants. Of these, 28 were due to liver failure, and 10 were due to HCC. Kaplan-Meier survival curves in ACLD (Figure 5) were significantly stratified by the presence of sarcopenia.

Prognostic factors in ACLD were analyzed. Univariate analysis based on the Cox proportional hazards model was performed for age, gender, BMI, etiology, blood parameters, LS, NLR, MELD-Na score, ALBI score, presence of ascites, sarcopenia, and HCC. Multivariate analysis (Table 5) with five significant factors selected by the stepwise method extracted ALBI score and sarcopenia as independent prognostic factors in ACLD.

**Table 5:** Prognostic factors by Cox proportional hazards model in ACLD. ALBI, albumin–bilirubin; CRP, C-reactive protein; HCC, hepatocellular carcinoma; LS, liver stiffness; MELD-Na, model for end-stage liver disease sodium; NLR, neutrophil-to-lymphocyte ratio; PT, prothrombin activity; TC, total cholesterol

	Univariate analysis		Multivariate analysis	
	HR (95%CI)	p-Value	HR (95% CI)	p-Value
Sex (male)	2.59 (1.25–6.06)	0.010		
Etiology (alcohol)	2.07 (1.03–3.98)	0.034		
Total bilirubin (mg/dL)	1.34 (1.16–1.50)	0.001		
Albumin (g/dL)	0.20 (0.13–0.30)	<0.001		
TC (mg/dL)	0.97 (0.97–0.98)	<0.001		
Hemoglobin (g/dL)	0.74 (0.63–0.86)	<0.001		
Platelets (x10^4^/mm^3^)	0.85 (0.80–0.91)	<0.001	0.93 (0.87–1.00)	0.057
CRP (≥0.5 mg/dL)	5.30 (2.80–10.13)	<0.001	1.60 (0.70–3.61)	0.264
PT (%)	0.95 (0.94–0.97)	<0.001		
Sodium (mEq/L)	5.54 (2.92–10.64)	<0.001		
Ammonia (μg/dL)	1.01 (1.01–1.02)	0.001		
NLR	1.31 (1.12–1.47)	0.002		
ALBI score	5.374(3.51–8.23)	<0.001	2.80 (1.49–5.28)	<0.001
MELD–Na score (≥15)	6.88 (3.54–13.12)	<0.001		
Ascites	12.16 (6.087–24.28)	<0.001	1.49 (0.57–3.92)	0.414
LS (kPa)	1.25 (1.16–1.33)	<0.001		
Sarcopenia	9.24 (4.31–22.87)	<0.001	3.44 (1.40–9.28)	0.006
Presence of HCC	4.56 (2.34–8.87)	<0.001		

## Discussion

This study aimed to investigate the association between liver disease-related sarcopenia and hepatic decompression in ACLD by analyzing MRE imaging data. The results showed that not only portal hypertension but also liver disease-related muscle mass loss occurs at LS ≥ 4 kPa in CLD. Additionally, the study establishes that sarcopenia in ACLD contributes to the early development of ascites and is linked to a poorer prognosis, independent of LS and liver severity.

MRE is currently regarded as the most accurate NIT for diagnosing and staging liver fibrosis, with the LS in healthy adults reported to be 2.10 kPa [29,30]. Recent reports indicate the utility of LS measurement by MRE in predicting liver-related events and assessing mortality risk in ACLD patients. In CLD, a decreased platelet count serves as a clinical indicator of liver disease progression, and thrombocytopenia acts as a simple indicator of portal hypertension, closely associated with the incidence and severity of gastroesophageal varices [31]. Based on our data, LSM of 4 kPa on MRE emerges as a potential initial reference value for predicting portal hypertension. The study aligns with the concept proposed at the Baveno VII consensus conference on portal hypertension [32], suggesting a stratification of liver complication risk using LS measured by transient elastography, where LS of 5 kPa is considered normal, and the risk increases with each additional 5 kPa. Our notion that LS ≥ 4 on MRE indicates a risk for portal hypertension seems consistent with this idea, implying that the risk occurs when LS exceeds twice the normal value. Ascites, the most common initial decompression event in cirrhosis, is highlighted in the study. Patients with grade 1 ascites exhibit significantly shorter survival than those without ascites [33,34]. The cut-off values of LS predicting hepatic decompensation on MRE have been investigated for various etiologies, ranging from 5.80 to 6.48 kPa [30,35-37]. Therefore, the clinical significance of the two identified landmarks on MRE (LS≥ 4 kPa and LS ≥ 6 kPa) in this study, encompassing multiple etiologies of ACLD, seems plausible.

The strength of this study is that sarcopenia in CLD could be assessed by MRE imaging data. Therefore, the timing of each diagnosis of LS by MRE and ascites/sarcopenia by MRI all coincided. Based on our data, sarcopenia was present in 29% of CLD, and age, BMI, ALD, systemic inflammation, and serum sodium level were independent associated factors. The prevalence of sarcopenia was significantly associated with higher LS. Age also showed a significant positive relationship with LS, but this relationship disappeared for LS ≥ 4 kPa, while %IGF-1 began to decrease significantly. Thus, our data suggest that the starting point of sarcopenia associated with liver disease is LS ≥ 4 kPa, and this event occurs at approximately the same time as the onset of portal hypertension. Portal hypertension is associated with malnutrition, the mechanisms of which include impaired digestion and absorption due to impaired intestinal peristalsis and edema, and insulin resistance [38]. In addition, portal hypertension causes systemic inflammation due to changes in the intestinal microbiota and bacterial translocation, which, together with hyperammonemia, leads to muscle wasting [13]. In addition, transjugular intrahepatic pressure shunting has been reported to increase muscle mass as a secondary effect in patients with cirrhosis [39]. Thus, the strong association between portal hypertension and sarcopenia in ACLD is undeniable, although several studies have shown that the hepatic venous pressure gradient itself does not correlate with muscle mass [40].

IGF-1 levels in healthy individuals are influenced by age, sex, and nutritional status, but it has been reported that patients with cirrhosis have decreased IGF-1 and increased GH levels due to positive feedback effects [10]. Thus, low IGF-1 is an important independent factor associated with age- and disease-related sarcopenia [11,41]. In this study, sarcopenic patients had significantly lower %IGF-1 levels than non-sarcopenic patients, and lower %IGF-1 levels were significantly associated with each item related to malnutrition, portal hypertension, and hepatic reserve function in CLD. Of particular note was the sharp decrease in %IGF-1 at LS ≥ 4 kPa, with a 20% difference in %IGF-1 in patients with LS < 4 kPa and those with LS of 4-5 kPa. The mechanism of low IGF-1 in cirrhosis is thought to be impaired IGF-1 synthesis in the liver, as well as hepatic GH resistance associated with liver dysfunction [10]. In a study investigating the response of IGF-I levels to GH replacement therapy in pediatric patients with CLD [42], it was reported that hepatic GH resistance is identified early in the disease process and becomes more pronounced as the disease progresses, especially in association with portal hypertension. Therefore, the present study suggests that the development of portal hypertension with LS ≥ 4 kPa in ACLD may also trigger a decrease in IGF-1, and these multiple factors may form the initial pathogenesis of muscle mass loss associated with liver disease.

Ascites serves as a crucial indicator in the natural progression of cirrhosis, often marking the initial manifestation of hepatic decompensation. Among patients with compensated cirrhosis, ascites is reported in 5-10% of cases annually, and its onset is associated with a notable decline in the five-year survival rate from 80% to 30% [43]. In the context of ACLD, this study identifies LS ≥ 6 kPa, ALBI score, and sarcopenia as independent factors linked to ascites presence. Notably, individuals with two or more of these factors concurrently face a high risk (96% of all ascites cases), with sarcopenia significantly expediting the development of ascites over a 24-month period in patients with high LS or mALBI grade 2b/3. Another important finding is that sarcopenia emerged as a poor prognostic factor in ACLD patients independent of liver reserve score, thus testing the hypothesis of this study. Recently, Dajti et al. [44] also reported that sarcopenia is an independent risk factor for ascites development and poor prognosis in cirrhotic patients based on a study in which sarcopenia was diagnosed by CT, which is consistent with our results.

On the other hand, our data raise a novel clinical question as to why sarcopenia promotes the development of ascites in ACLD. In the present study, low serum sodium was an independent factor associated with sarcopenia in CLD. Many studies have shown that dilutional hyponatremia is common in patients with cirrhosis and is associated with ascites and decreased survival [45,46]. However, while the mechanisms of hyponatremia in decompensated cirrhosis are well understood, little work has been done in the pre-ascites stage of cirrhosis [47,48]. The AASLD (American Association for the Study of Liver Diseases) Practice Guidance [49] classifies LS of 3-4 kPa on MRE as compensated ACLD (cACLD), and in our previous study [50], LS > 3.4 kPa in CLD patients was associated with portal hypertension, low serum sodium levels, and a high risk of sarcopenia. Thus, it is speculated that the presence of sarcopenia in cACLD may be an a priori stage of hepatic decompensation failure associated with dilutional hyponatremia. However, further studies are needed to prove this.

There are several limitations in the present study. This study is a single-center retrospective analysis and needs to be validated at other centers. In particular, the diagnostic criteria for sarcopenia vary among reporters, and our criteria for sarcopenia were set based on low BMI values, which reflect malnutrition. Therefore, the reproducibility of our results in other sarcopenia criteria needs to be investigated. Second, the type of hyponatremia is not demonstrated to be dilutional in the present study. We based our discussion of the present study on the assumption that most hyponatremia cases in ACLD are hypervolemic or dilutional.

## Conclusions

In conclusion, both portal hypertension and liver disease-related sarcopenia in CLD manifest at LS ≥ 4 kPa as measured by MRE, suggesting a strong association between these two conditions. Additionally, the presence of sarcopenia in cACLD is linked to an earlier onset of ascites, likely contributing to the poor prognosis in ACLD.
